# Ferroelectric-Based Optoelectronic Synapses for Visual Perception: From Materials to Systems

**DOI:** 10.3390/nano15110863

**Published:** 2025-06-04

**Authors:** Yuqing Hu, Yixin Zhu, Xinli Chen, Qing Wan

**Affiliations:** 1 Yongjiang Laboratory, Functional Materials and Devices Heterogeneous Integration Research Center, Ningbo 315201, China; yuqing-hu@ylab.ac.cn (Y.H.); yixin-zhu@ylab.ac.cn (Y.Z.); xinli-chen@ylab.ac.cn (X.C.); 2 School of Microelectronics, University of Science and Technology of China, Hefei 230026, China

**Keywords:** ferroelectric, polarization regulation, artificial visual perception, bionic synapses, neuromorphic computing

## Abstract

More than 70% of the information humans acquire from the external environment is derived through the visual system, where photosensitive function plays a pivotal role in the biological perception system. With the rapid development of artificial intelligence and robotics technology, achieving human-like visual perception has attracted a great amount of attention. The neuromorphic visual perception system provides a novel solution for achieving efficient and low-power visual information processing by simulating the working principle of the biological visual system. In recent years, ferroelectric materials have shown broad application prospects in the field of neuromorphic visual perception due to their unique spontaneous polarization characteristics and non-volatile response behavior under external field regulation. Especially in achieving tunable retinal neural synapses, visual information storage processing, and constructing dynamic visual sensing, ferroelectric materials have shown unique performance advantages. In this review, recent progress in neuromorphic visual perception based on ferroelectric materials is discussed, elaborating in detail on device structure, material systems, and applications, and exploring the potential future development trends and challenges faced in this field.

## 1. Introduction

More than 70% of the information obtained by humans from the external world comes from the visual system, which means that the photosensitive function plays a crucial role in the biological perception system [1]. The retina converts external light information into electrical signals and transmits them to the visual center through neurons; then they are processed and remembered by the brain [2]. With the development of humanoid robots, researchers have shown a strong interest in how to achieve visual biomimetics. Photodetectors can rapidly respond to optical signals and convert them into electrical signals [3,4,5]. However, the electrical signal quickly decays to its initial state while the optical stimulus is removed, which means that the photodetector lacks the capability for nonvolatile optical information storage. In contrast, optoelectronic synapses integrate light sensing and synaptic functions [6]. It can not only respond to light stimuli but also achieve real-time processing and temporary storage of optical information in parallel [7,8]. This working mode effectively eliminates unnecessary consumption, which is very similar to the human visual system. In addition, when optoelectronic synapses are used in neuromorphic computing, the devices can effectively simulate the structure and function of the human brain.

The operating principle of optoelectronic synaptic devices is based on the photogenerated carrier dynamics in semiconductor materials. The underlying mechanisms primarily involve carrier trapping and release processes at defect states or heterojunction interfaces [9,10], photo-induced phase transition [11,12,13], and persistent photoconductivity effects in semiconductors [14,15]. These devices exhibit nonvolatile resistive/current responses in external circuits under optical pulse stimulation. Therefore, regulating the transport and recombination process of photogenerated carriers to adjust the internal resistance of the device is the key to achieving biological synaptic functional behavior. For optoelectronic synapse devices, the relaxation characteristics of the device are important indicators for measuring its performance [16]. The optoelectronic synapse based on a single-layer semiconductor has the advantage of simple device structure, but its ability to change the relaxation characteristics of optoelectronic synapse devices is limited [17]. To control the relaxation characteristics and responsiveness of the device, researchers adjusting the composition of the oxide semiconductor has been suggested [18,19]. However, the realization of tunable multi-relaxation states in individual devices remains challenging using existing methodologies, which has motivated researchers to explore novel device architectures. Initially, a dielectric layer is introduced to modulate the charge density at the semiconductor/dielectric interface through the application of a gate bias (V_G_), thereby controlling device performance [20,21]. However, the requirement of V_G_ inevitably increases power consumption of devices. In this regard, semiconductor/ferroelectric heterostructure devices emerge as a promising alternative for achieving tunable optoelectronic synaptic functionalities, owing to their unique polarization characteristics [22].

Ferroelectric materials exhibit multi-stable states due to the reversible polarization switching of ferroelectric domains under electric fields, which provides a basis for simulating weight modulation of biological synapses [23,24,25]. In optoelectronic synaptic devices, the semiconductor/ferroelectric heterostructure dynamically modulates the optoelectronic properties through ferroelectric polarization field effects [26]. The depolarization field caused by the polarization of ferroelectric materials can bend the energy band at the interface, thereby regulating the recombination efficiency of photogenerated charge carriers [27]. In addition, the polarized interface related to ferroelectric polarization also serves as controllable capture centers, achieving the bound of photogenerated carriers and causing changes in the relaxation behavior of synaptic devices [26,28]. These devices, building upon these principles, can not only simulate biological synapse-like behaviors but also combine optical sensing with memory functions [29,30,31]. In addition, these devices fundamentally overcome the von Neumann bottleneck by integrating optical sensing, nonvolatile memory, and in-memory computing capabilities. This lays the foundation for building integrated sensing-memory-computing systems in neuromorphic engineering.

**Figure 1:**
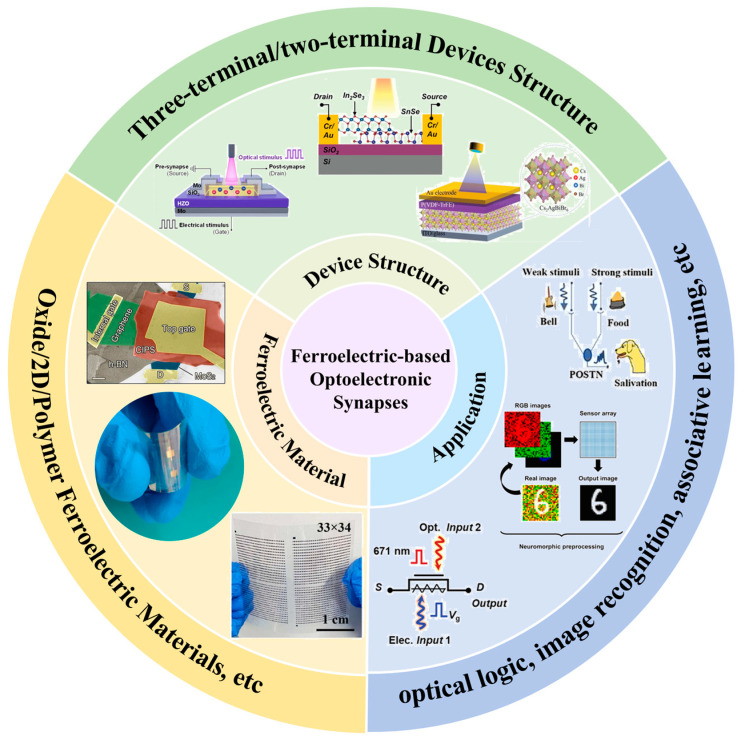
Overview of this Review. The three aspects of device structure, selection of ferroelectric material, and application are specifically included [32,33,34,35,36,37,38,39]. Reprinted with permission from [40]. Copyright 2022 American Chemical Society.

This review focuses on ferroelectric-material-based optoelectronic synapses, encompassing both two-terminal and three-terminal device configurations. Diverging from previous reviews concentrating on specific material systems or device architectures, we particularly highlight the selection of ferroelectric materials and their applications in bio-inspired vision systems for neuromorphic computing. Firstly, the neurobiological principles of visual information processing were introduced. Then, various types of ferroelectric optoelectronic synapse devices were systematically summarized, and their structural design and operational mechanisms were discussed. Listed the applications of different types of ferroelectric materials in devices and their implementation in visual perception and neural morphology computing. Finally, an overview of the current challenges and future research directions in this emerging field was provided. The overview of this review is shown in Figure 1.

## 2. The Principle and Structure of Ferroelectric-Based Optoelectronic Synapse Devices

**Figure 2:**
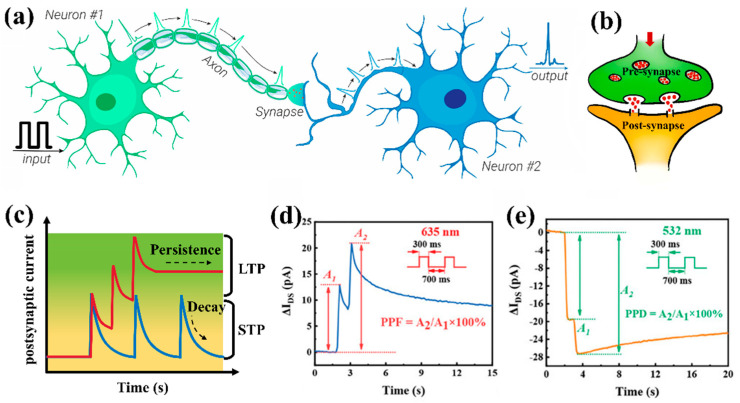
Schematic diagram of (**a**) biological neuronal system [41] and (**b**) synapse [55]. (**c**) Changes in postsynaptic currents with long-term and short-term plasticity over time. (**d**) Paired-pulse facilitation and (**e**) paired-pulse depression behavior in artificial synapses [51].

Prior to in-depth discussion, it is essential to comprehend the fundamental functions of synapses and their validation methods in artificial synaptic devices. As the most structurally and functionally complex organ in the human body, the brain contains approximately 10^11^ neurons interconnected through an estimated 10^15^ synapses [41,42]. Neurons, composed of synaptic terminals (dendrites and axons) and cell bodies, constitute the basic functional units of neural circuits [43]. Figure 2a shows the schematic diagram of two neurons connected by synapses. In neural systems, neurons generate action potentials under excitatory or inhibitory states, transmitting signals to subsequent neurons via synaptic connections through membrane potential differences caused by ionic concentration gradients across cellular membranes [44]. Figure 2b schematically shows the structure of a biological synapse. The electrical spikes generated during signal transmission to presynaptic neurons are termed action potentials. These transient potential alterations modulate ionic concentrations around postsynaptic membranes, enabling continuous generation and propagation of subsequent potential signals [45]. The resultant potential signals on postsynaptic membranes, induced by presynaptic stimulation, are defined as postsynaptic currents. These synaptic connections exhibit cumulative effects, with postsynaptic currents being categorized into excitatory postsynaptic currents (EPSC) or inhibitory postsynaptic currents (IPSC) based on their potentiating or depressing characteristics [46,47]. The dynamic variation of postsynaptic currents, referred to as synaptic plasticity, plays a pivotal role in biological information processing and neural signal transmission.

As illustrated in Figure 2c, synaptic plasticity can be further classified into short-term plasticity (STP) and long-term plasticity (LTP) based on the duration of postsynaptic current retention (information retention time) [41]. In the human brain, short-term memory typically exhibits transient retention with negligible current-level variations, indicating that the recognized information is prone to rapid decay [48,49]. Conversely, long-term memory plasticity demonstrates sustained current-level modifications that enable prolonged information retention, which is considered fundamental for cognitive processes ranging from basic classical conditioning to advanced higher-order thinking [50]. Figure 2d,e presents two characteristic biological synaptic phenomena: paired-pulse facilitation (PPF) and paired-pulse depression (PPD) [51]. The facilitation (or depression) degree can be quantified by the *A*_2_/*A*_1_ ratio (PPF/PPD index), where *A*_1_ and *A*_2_ represent the current peak amplitudes elicited by the first and second pulse stimuli, respectively. Furthermore, the transition from STP to LTP can be achieved through modulated pulse stimulation, thereby enhancing information transmission efficiency. Additional critical parameters for characterizing synaptic devices include spike-duration-dependent plasticity (SDDP), spike-rate-dependent plasticity (SRDP), and spike-number-dependent plasticity (SNDP), which collectively describe the dynamic response characteristics of synaptic devices under various stimulation paradigms [52,53,54].

Synaptic plasticity serves as the molecular foundation for biological learning and memory. To emulate visual mechanisms, optoelectronic devices with synaptic functionalities can be employed, which are pivotal for realizing low-power artificial vision systems with neuromorphic computing capabilities [8,56,57]. This section focuses on two main optoelectronic synapse device structures based on ferroelectric materials: three-terminal and two-terminal devices.

### 2.1. Three-Terminal Ferroelectric Field-Effect Transistor Synaptic Devices

#### 2.1.1. Basic Device Structure and Operating Principle

The multi-material stacking structure of three-terminal ferroelectric field-effect transistor (FeFET) synaptic devices allows them to easily combine functional materials, such as optoelectronic materials and achieve biological simulation of synaptic function by precisely adjusting the conductivity of channels [58,59]. These devices utilize electric field-induced ferroelectric domain reorientation in ferroelectric materials to enable multilevel tuning of carrier concentration in the semiconductor channel, thereby storing synaptic weight information in nonvolatile polarization states. Under an applied gate electric field, the electric dipoles in ferroelectric materials undergo aligned orientation, forming specific nonvolatile domain configurations (e.g., upward/downward polarization or intermediate states). The polarized charges of ferroelectric domains induce screening charges at the ferroelectric/semiconductor interface, thereby modulating the carrier concentration in the semiconductor channel. Furthermore, the ferroelectric polarization field can modify the band bending at the interface, enabling tunable Schottky barrier heights. Collectively, these mechanisms facilitate nonvolatile multilevel conductance modulation in the semiconductor channel.

**Figure 3:**
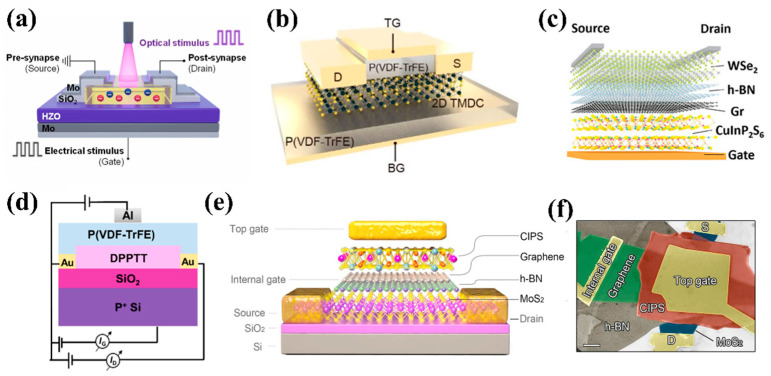
(**a**) Schematic illustration of bottom-gate FeFET based on HZO [61]. (**b**) Schematic illustration of dual-gate FeFET based on P(VDF-TrFE). Reprinted with permission from [32]. Copyright 2022, American Chemical Society. (**c**) Schematic illustration of bottom-gate FeFET based on CuInP_2_S_6_ [62]. (**d**) Schematic diagram of top-gate FeFET based on P(VDF-TrFE) [63]. (**e**) Schematic configuration and (**f**) SEM image of an MoS_2_/h-BN/graphene/CIPS vdW FeFET [33].

Optoelectronic synapse devices with FeFET structure can usually be divided into bottom-gate and top-gate structures. FeFETs with bottom gate structures have fewer limitations on the selection of ferroelectric materials; whether oxide ferroelectric materials, flexible polymer ferroelectric materials, or emerging two-dimensional ferroelectric materials, all can be used as gate dielectrics [32,60,61,62]. Figure 3a–c show bottom-gate FeFETs based on Zr-doped HfO_2_ (HZO) thin film, Polyvinylidene Fluoride-Trifluoroethylene (PVDF-TrFE), and CuInP_2_S_6_ (CIPS) ferroelectric materials, respectively. In contrast, the top-gate structure imposes strict requirements on the selection of ferroelectric materials, which requires ensuring the quality of the ferroelectric layer while also preventing the semiconductor layer from being damaged by subsequent processes. Therefore, it is usually necessary to use low damage deposition processes (such as spin coating or van der Waals transfer technology) to prepare polyvinylidene fluoride-based (PVDF-based) polymers or van der Waals (vdW) layered materials (such as CIPS), as shown in Figure 3d–f [33,63]. These ferroelectric materials can form low-defect-density ferroelectric/semiconductor interfaces without relying on epitaxial growth, thereby ensuring the stability of polarization flip and precise control of synaptic weights. These devices achieve synaptic weight modulation through three basic physical processes: Precise control of ferroelectric domain states through gate voltage parameters (pulse width/amplitude/frequency); Band bending modulation at the semiconductor interface via depolarization fields; Regulation of photogenerated carrier separation and recombination kinetics in the semiconductor. The detailed principle will be introduced in the third part in conjunction with specific ferroelectric materials.

#### 2.1.2. Advantages and Challenges

FeFET exhibits unique advantages in optoelectronic synapse applications due to its excellent high-density integration potential and good compatibility with CMOS processes [64,65]. In addition, its excellent durability and ultra-low operating energy consumption in the sub-picojoule range make it an ideal choice for large-scale neural morphological computing systems [66]. From the perspective of working mechanism, FeFET achieves low-noise synaptic weight updating and non-volatile information storage by precisely regulating the channel conductivity through gate voltage. As mentioned earlier, the key to the regulation of synaptic weights in FeFET devices by the ferroelectric layer lies in the steady-state characteristics of the ferroelectric domains. When the channel length is maintained above 100 nm, a stable multi-domain structure can be formed in the ferroelectric layer, and progressive threshold voltage modulation can be achieved through domain wall motion, thus perfectly simulating the continuous weight changes of biological synapses [23,67]. However, the inevitable transition to single-domain states critically disrupts this mechanism, as the loss of mobile domain walls forces binary polarization switching characterized by abrupt, all-or-nothing transitions. Such discrete behavior reduces the conductance modulation resolution from multiple levels to merely two states, ultimately degrading neuromorphic computing performance [24].

To address this issue, researchers have proposed multiple solutions: In terms of device architecture, three-dimensional integration technology (such as back-end-of-line (BEOL) FeFET) is used to expand the effective gate area [68,69]. At the system level, construct a randomly connected multi-FeFET array network [69]. These innovative strategies not only successfully broke through the performance limitations of traditional FeFET but also laid a solid foundation for the development of a new generation of optoelectronic synapse devices that integrate environmental perception, information storage, and signal processing, pushing the practical application process of neuromorphic visual systems to new heights.

### 2.2. Two-Terminal Ferroelectric Synapses with Vertical Structure

#### 2.2.1. Basic Device Structure and Operating Principle

Vertical two-terminal optoelectronic synapse devices adopt a metal-functional layer-metal vertically stacking architecture, where the functional layer is usually composed of heterostructures formed by photosensitive semiconductor and ferroelectric material or a single layer of ferroelectric semiconductor material [34,55,70,71]. Under optical stimulation, photons excite electron-hole pairs in the semiconductor layer, which are efficiently separated via built-in electric fields (such as Schottky barriers or ferroelectric polarization fields), generating photocurrents. Meanwhile, the non-volatile polarization state of the ferroelectric layer can be controlled by an external electric field to dynamically alter the band bending and carrier transport behavior at the semiconductor interface, thereby achieving multi-level storage and updating of synaptic weights.

**Figure 4:**
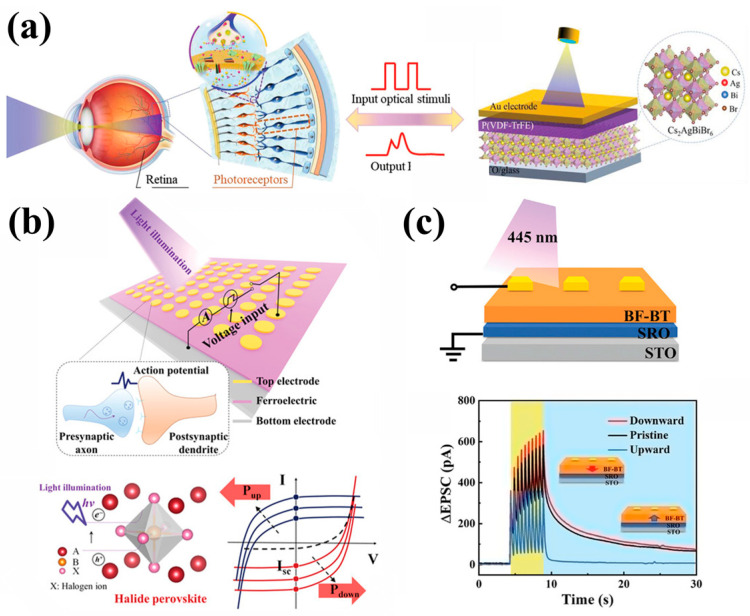
(**a**) Schematic diagrams of human visual system and the diagram of photonic synapse based on Au/P(VDF-TrFE)/Cs_2_AgBiBr_2_/ITO heterostructure [34]. (**b**) The schematic illustration of the photo-ferroelectric synapse with electric writing and photocurrent reading, halide perovskite under light illumination, and varying I_sc_ with the switching ferroelectric polarization [71]. (**c**) ΔEPSC changes of synapse devices under different polarization states of BF-BT [55].

For example, Figure 4a demonstrates a biomimetic optoelectronic synapse based on Au/P(VDF-TrFE)/Cs_2_AgBiBr_2_/ITO heterostructure. Lao et al. [34] utilized P(VDF-TrFE) to introduce energy potential wells for hole carriers at the ferroelectric/semiconductor interface, prolonging the carrier lifetime and effectively simulating biological synaptic behavior. Researchers have also conducted related studies on the implementation of tunable optoelectronic synapses based on single-layer functional layers. As shown in Figure 4b, the organic-inorganic halide perovskite ((R)-(-)-1-cyclohexylethylammonium)PbI_3_(R-CYHEAPbI_3_) photo-ferroelectric serves as the functional layers; the organic layer provides ferroelectric dipole, and the PbI_6_ octahedron is responsible for photon absorption and charge transport [71]. In addition, the preparation of optoelectronic synapse devices can also be achieved in single-layer oxide ferroelectric semiconductors, as shown in Figure 4c [55]. In BiFeO_3_-BaTiO_3_(BF-BT) ferroelectric semiconductor-based devices, the flipping of ferroelectric domains can regulate the recombination rate of photogenerated carriers, achieving tunable synaptic behavior simulation effectively. These architectures not only achieve ultralow power operation but also provide a versatile platform for neuromorphic computing by seamlessly integrating optical sensing, memory, and processing functionalities.

#### 2.2.2. Advantages and Challenges

Vertical two-terminal optoelectronic synaptic devices have emerged as promising candidates for neuromorphic computing systems, offering several compelling advantages over conventional architectures. These devices feature a simple yet efficient structure with remarkable potential for high-density integration (4F^2^ unit area) and ultrafast switching speeds at the nanosecond scale. Significantly, certain devices operate in photovoltaic mode, enabling functionality at zero bias with ultralow energy consumption as minimal as sub-picojoule per spike [34]. The vertical device architecture not only provides an ultracompact footprint but also offers excellent potential for three-dimensional stacking, making it highly suitable for large-scale neuromorphic integration. In addition, these optoelectronic synapses demonstrate unique capabilities in direct optoelectronic coupling, enabling simultaneous environmental perception, information storage, and signal processing within a single device.

Despite these advantages, several critical challenges must be addressed to realize the full potential of this technology. Material stability remains a primary concern, as perovskite-based devices are susceptible to degradation under environmental stressors such as moisture and thermal fluctuations. Interface engineering presents another significant challenge, with defect states at ferroelectric/semiconductor heterojunctions leading to non-radiative recombination and compromised device uniformity. Furthermore, scaling below 100 nm introduces fundamental limitations, as ferroelectric mono-domain formation both in two-terminal and three-terminal configurations disrupts the essential multi-state storage capability. It can be expected that the development of new narrow bandgap ferroelectric semiconductors, implementation of advanced interface passivation strategies, and optimization of three-dimensional integration processes will greatly promote the practical application of the next generation of machine vision systems.

### 2.3. Two-Terminal Ferroelectric Synapses with Parallel Structures

#### 2.3.1. Basic Device Structure and Operating Principle

Horizontal two-terminal optoelectronic synaptic devices have demonstrated remarkable progress in achieving deep integration of optoelectronic signals with neuromorphic computing through ferroelectric p-n heterojunctions and molecular ferroelectric/semiconductor interface structures [8,30,31,35]. The working principle of these two structures is both based on the non-volatile switching of ferroelectric polarization. Relying on the built-in electric field or interface potential barrier changes caused by ferroelectric polarization to achieve control over photocurrent or conductivity characteristics.

**Figure 5:**
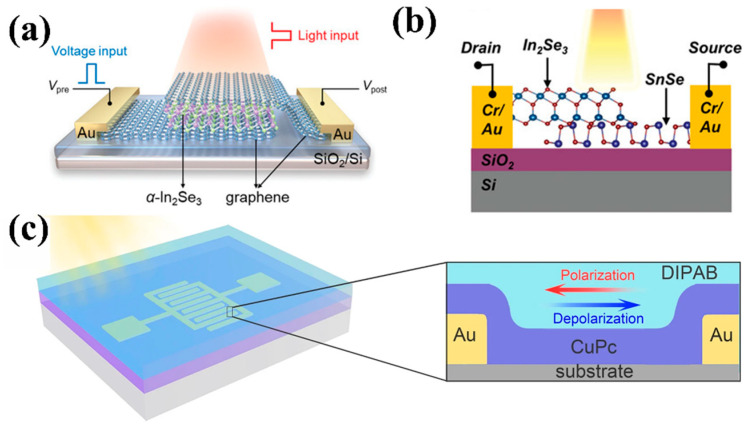
Schematic structure of the (**a**) graphene/α-In_2_Se_3_/graphene [31] and (**b**) α-In_2_Se_3_/SnSe [35] ferroelectric p-n junction device. (**c**) 3D and cross-sectional structure of the interfacial device based on DIPAB ferroelectric [27].

As shown in Figure 5a, Wang et al. [31] demonstrated an optoelectronic synaptic device based on a graphene/α-In_2_Se_3_/graphene crossbar structure. The polarization of ferroelectric semiconductor α-In_2_Se_3_ can effectively adjust the Schottky barrier height of the interface, thereby enabling reconfigurable device conductance and photoresponsivity. Similarly, a two-terminal 2D ferroelectric α-In_2_Se_3_/SnSe p–n heterojunction is illustrated in Figure 5b [35]. Based on the co-modulation of the polarization reconfigurable built-in electric field caused by the p-n junction and the photo-induced ferroelectric polarization switch, visual synaptic function was successfully simulated in the device. Cai et al. [27] constructed a molecular ferroelectric (MF)/semiconductor interface, utilizing the high ferroelectricity and polarization field amplification effect of MF to precisely regulate carrier injection in the semiconductor layer, thereby adjusting synaptic weight. The schematic diagram of the diisopropylammonium bromide (DIPAB)/Copper phthalocyanine (CuPc) heterojunction device is depicted in Figure 5c. These systems achieve multilevel resistive switching through polarization-dependent carrier blocking effects, demonstrating another pathway for implementing synaptic functionality in optoelectronic devices.

#### 2.3.2. Advantages and Challenges

Horizontal two-terminal optoelectronic synapse devices based on two-dimensional materials have unique advantages of atomic-scale accuracy and are not limited by lattice matching. These devices combine unprecedented integration density through their simplified architecture with full CMOS compatibility while achieving multifunctional operation that integrates optical sensing, nonvolatile memory, and synaptic plasticity in a single unit cell. The van der Waals nature of 2D materials enables the creation of ultra-clean interfaces that facilitate remarkable performance metrics, including synaptic plasticity with high PPF index and wavelength-selective responses that emulate biological visual processing [31,35]. Particularly noteworthy is their ability to maintain these characteristics while operating at ultralow power levels, a critical advantage for energy-efficient neuromorphic systems.

However, to fully unleash its potential, there are still some issues that need to be overcome. Synthesizing large-area, high-quality 2D ferroelectric semiconductors with uniform polarization properties at wafer scale remains a significant materials challenge, while interface engineering requires precise control to minimize defect-mediated recombination losses. In addition, the polarization fatigue and limited spectral response range of two-dimensional materials limit their practical deployment. By developing methods for preparing large-sized 2D ferroelectric materials with controllable bandgap, improving material stability is expected to promote the development of two-dimensional ferroelectric optoelectronic synapse devices towards high performance and reliability.

## 3. Ferroelectric Materials for Optoelectronic Synapses

The performance of ferroelectric-based optoelectronic synapse devices depends largely on the selection of ferroelectric materials. The characteristics of ferroelectric materials directly affect device performance indicators: polarization strength determines the dynamic range of synaptic weight updates, bandgap structure affects light absorption efficiency, and interface compatibility determines charge carrier transport and device reliability [23,72,73]. Especially in devices where the ferroelectric layer serves as the gate dielectric, the coercive field of the ferroelectric material fundamentally determines the operating voltage and energy consumption of the device. For example, HfO_2_-based ferroelectrics with low coercive fields can perform synaptic operations at the sub-picojoule level [74,75]. Two-dimensional ferroelectric semiconductors (such as α-In_2_Se_3_) achieve deep coupling between light absorption and ferroelectric modulation through their atomic-scale thickness and in-plane polarization [76,77]. While polymer ferroelectric materials (such as PVDF-based ferroelectrics) provide a new option for flexible devices through molecular dipole orientation [78,79]. It is worth noting that ferroelectric polarization regulates the ability of the photo-induced carrier relaxation process. Multi-timescale synaptic plasticity from milliseconds to non-volatile can be achieved by suppressing interface recombination or inducing defect state redistribution through polarization fields. Therefore, this section will systematically discuss the relationship between the selection optimization of three main ferroelectric material systems (oxide ferroelectric, two-dimensional ferroelectric, and polymer ferroelectric) in ferroelectric-based optoelectronic synapses and device performance, providing theoretical guidance for the selection of functional materials in neural morphology computing applications.

### 3.1. Oxide Ferroelectric Materials

**Figure 6:**
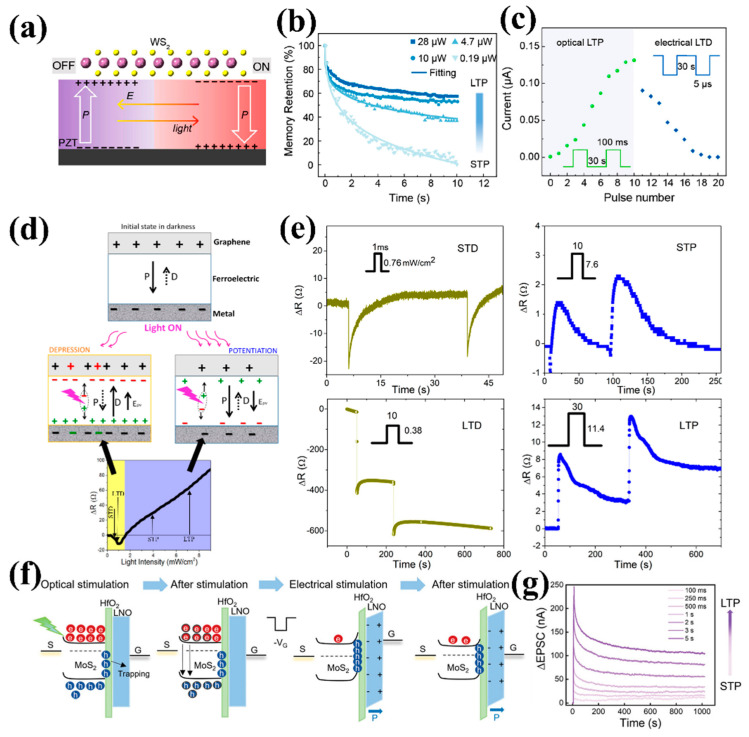
(**a**) Schematic configuration of the mechanism behind the optically and electrically tunable channel conductance of WS_2_/PZT optoelectronic synapses. (**b**) Different intensities of light illumination trigger STP to LTP behaviors. (**c**) Long-term optical potentiation and electrical depression in the device. Reprinted with permission from [26]. Copyright 2020 American Chemical Society. (**d**) Model the competition between photovoltaic (PV) and pyroelectric effects under light stimulation. (**e**) The implementation of four basic functions: STD, STP, LTD, and LTP. Reprinted with permission from [83]. Copyright 2023 American Chemical Society. (**f**) Mechanism illustration of optical/electrical modulation in the LiNbO_3_/HfO_2_/MoS_2_ ferroelectric transistors. (**g**) STP-LTP transition under light stimulation with different pulse widths [22].

Oxide ferroelectric materials typically exhibit high saturation polarization strength, high Curie temperature, excellent dielectric constant, and low dielectric loss [80]. These outstanding characteristics enable oxide ferroelectric materials to exhibit strong modulation for semiconductors, giving them unique advantages for optoelectronic synapse applications [81,82]. For example, a study demonstrated an optoelectronic synaptic device based on a two-dimensional WS_2_/PZT FeFET structure, which achieved integrated sensory-memory functionality mimicking biological visual systems through optoelectronic cooperative modulation [26]. As shown in Figure 6a, the PZT thin film below WS_2_ undergoes ferroelectric polarization reversal downwards induced by light, while polarization reversal upwards occurs under the action of an electric field. Figure 6b shows the protrusion behavior of the device under different light intensity stimuli. Under 532 nm optical pulse excitation, WS_2_ generates photoinduced charges that alter the polarization orientation of PZT, thereby modulating channel conductance to successfully simulate the STP-LTP transition. The light-driven ferroelectric polarization switching arises from the interplay between photoinduced charges in the 2D semiconductor and polarization charges in the ferroelectric layer. Specifically, the WS_2_/PZT heterostructure exhibits a preferential downward polarization, resulting in a built-in electric field (E_bi_) oriented downward. When the PZT is initially in the upward-polarized state, optical absorption in WS_2_ generates intralayer excitons, which subsequently decay into interlayer excitons, leading to the accumulation of positive charges at the WS_2_/PZT interface. These photoinduced charges effectively screen the upward polarization, triggering an E_bi_-driven reversal of the ferroelectric polarization. The device exhibits distinct memory behaviors: low-light-intensity-induced STP shows rapid conductance decay, while high-intensity illumination triggers LTP with sustained conductance enhancement. This intensity-dependent STP-LTP transition faithfully replicates the “multi-storage model” of human memory. Furthermore, through electrically induced ferroelectric domain reversal, the device achieves long-term depression (LTD), completing the full spectrum of synaptic plasticity regulation (Figure 6c).

For instance, Maity et al. [83] developed a simplified all-optical synaptic control scheme by constructing a graphene/ferroelectric PMN-PT single-crystal heterostructure, achieving complete optical modulation of LTP and LTD using single-wavelength (365 nm) light pulses. Figure 6d visualizes the working principle of the device, which is the competition between photovoltaic (PV) and pyroelectric effects in PMN-PT. Under low light intensity, the pyroelectric effect dominates, inducing ferroelectric depolarization that reduces graphene resistance (STD/LTD). Conversely, the PV effect prevails under high intensity, where photogenerated charges reorient ferroelectric polarization to increase resistance (STP/LTP). As shown in Figure 6e, the intensity-dependent bidirectional modulation not only accurately replicates biological plasticity but also demonstrates millisecond-scale response speeds—two orders of magnitude faster than biological synapses.

Although lead-based ferroelectric materials demonstrate excellent performance in optoelectronic synaptic devices, researchers have increasingly turned to lead-free alternatives due to tightening environmental regulations (e.g., the RoHS Directive) and toxicity concerns [84,85]. Wang et al. [22] reported a highly controllable optoelectronic device based on LiNbO_3_/HfO_2_/MoS_2_ ferroelectric transistors. Figure 6f illustrates the working principle of the device under optical signal (532 nm) or electrical signal (gate voltage). After applying a light signal, the electron-hole pairs in the optically excited channel layer separate. Due to the confinement of some charge carriers by the LiNbO_3_ ferroelectric layer, after removing the light, the current will remain in a higher current state than the initial current state. The device utilizes 532 nm optical pulses to induce interfacial charge trapping for low-resistance state (LRS) switching, while electrical pulses restore the high-resistance state (HRS) through ferroelectric polarization modulation, demonstrating excellent non-volatile memory characteristics. Due to the initial adjustable output current state, the FeFET successfully emulates various synaptic behaviors, including STP and LTP, as shown in Figure 6g.

**Figure 7:**
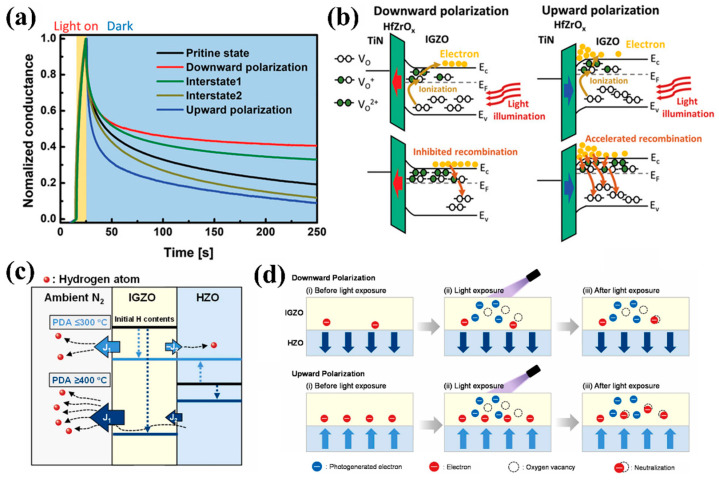
(**a**) The relaxation behaviors of devices under different polarization states of HfZrO_x_. (**b**) Schematic diagram of IGZO/HfZrO_x_ interface band changes under different polarizations [81]. (**c**) Schematic of proposed dynamics of H atoms during the PDA process. (**d**) Schematic illustration of photo-current decay mechanism in HZO/IGZO FeFET with downward/upward polarization [61].

In recent years, the unique combination of CMOS-compatible fabrication, nanoscale ferroelectric stability, and sub-picojoule energy consumption has established hafnium-based ferroelectric materials as an ideal candidate for next-generation neuromorphic computing devices [66,86,87]. Kim et al. [81] introduced HrZrO_x_ ferroelectric thin film as a gate to regulate the relaxation characteristics of the device. Through the pre-application of bias pulses with varying polarity and magnitude to the gate electrode, four distinct polarization states were induced in the ferroelectric layer: downward polarization, interstate 1, interstate 2, and upward polarization. As shown in Figure 7a, according to the different polarization states of the ferroelectric layer, indium gallium zinc oxide (IGZO) exhibits significantly different relaxation behaviors. This phenomenon originates from polarization-induced band bending at the IGZO/HfZrO_x_ interface, which modulates electron depletion or accumulation. The corresponding mechanisms are depicted in Figure 7b. Under downward polarization, electrons are depleted at the interface, and the separation between photogenerated electrons and ionized oxygen vacancies can suppress recombination of photogenerated charge carriers to prolong relaxation time. On the contrary, upward polarization induces electron accumulation at the interface, which promotes the recombination reactions. Thus, a photon synapse with tunable synaptic function was successfully prepared based on the above mechanism.

Recently, researchers have successfully achieved synergistic modulation between the electrical properties of IGZO channels and the ferroelectric phase of HZO through optimized post-deposition annealing (PDA) processes, enabling high-performance IGZO/HZO FeFET [61]. As shown in Figure 7c, the PDA temperature (400–600 °C) significantly affects device performance by regulating mechanisms such as hydrogen (H) migration, oxygen vacancy (Vo) formation, and IGZO film densification. At the optimized 600 °C annealing condition, the 10 nm-thick HZO layer demonstrates a stabilized orthorhombic ferroelectric phase with significantly enhanced remnant polarization (P_r_), while simultaneously achieving expected conductivity of the IGZO channel. The resulting FeFETs exhibit a memory window of ~1 V and endurance exceeding 10^4^ cycles. Figure 7d showcases a schematic illustration of the photo-current decay mechanism in HZO/IGZO FeFET. The polarization state of the HZO layer significantly affects the accumulation or depletion of electrons at the IGZO/HZO interface, thereby affecting the photocurrent attenuation rate of the device. This enables the device to emulate bio-synaptic functions through electro-optical dual-mode modulation.

### 3.2. Two-Dimensional Ferroelectric Semiconductor Materials

Van der Waals ferroelectric materials have the ability and the capability for on-demand arbitrary stacking without lattice-matching constraints, enabling unprecedented design freedom for atomically precise heterostructures, providing a foundation for constructing ultra-small programmable optoelectronic synapse devices [62,88]. Crucially, the in-plane/out-of-plane polarization in vdW ferroelectrics can be effectively modulated by multiple external stimuli, including light illumination and electric fields, making them ideal platform materials for constructing ultrasmall programmable optoelectronic synaptic devices [89,90,91]. Among the developed 2D ferroelectric semiconductors, α-In_2_Se_3_ has attracted significant attention due to its unique room-temperature ferroelectricity and excellent photoelectric response [92,93]. This material exhibits both in-plane and out-of-plane spontaneous polarization with a ferroelectric phase transition temperature exceeding 320 K. Another representative material, CuInP_2_S_6_ (CIPS), exhibits a unique interlayer slip ferroelectric mechanism with a Curie temperature of ~315 K [94,95,96]. These 2D semiconductor ferroelectric materials set up new avenues for the development of next-generation neuromorphic optoelectronic devices.

**Figure 8:**
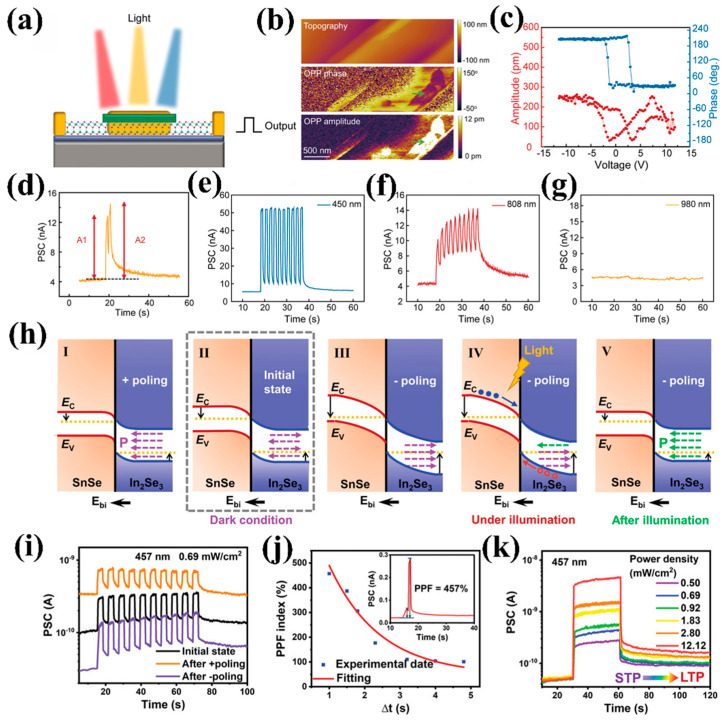
(**a**) Schematic diagram of optoelectronic synapse device. (**b**) Topography, OOP PFM phase, and OOP PFM amplitude images of α-In_2_Se_3_. (**c**) PFM phase (blue line) and PFM amplitude (red line) hysteresis loops of α-In_2_Se_3_. (**d**) PPF behavior of devices based on α-In_2_Se_3_/GaSe ferroelectric 2D van der Waals heterojunction. The photoelectric responses of the devices at wavelengths of (**e**) 450, (**f**) 808, and (**g**) 980 nm, respectively [97]. (**h**) The energy band diagrams and corresponding variations of built-in potentials under different polarization and illumination states. (**i**) Synaptic behavior of devices under different polarization states. (**j**) PPF index as a function of optical pulse interval time ∆t. (**k**) STP-LTP transition of the devices [35].

As shown in Figure 8a, Guo et al. [97] proposed a multifunctional optoelectronic synapse device based on α-In_2_Se_3_/GaSe ferroelectric 2D van der Waals heterojunction, which integrates optical/electrical stimulation response with ferroelectric polarization regulation to achieve full functional simulation of the human visual system (retina, optic nerve, and visual cortex) in a single device. The α-In_2_Se_3_ ferroelectric channel exhibits out-of-plane (OOP) polarization (verified by PFM hysteresis loops, as shown in Figure 8b,c) and a narrow 1.4 eV bandgap, enabling efficient photoelectric conversion (52 nA photocurrent at 450 nm) and low-power LTP/LTD modulation (±3 V). In addition, the device exhibits good PPF behavior under light pulse stimulation, and the PPF index can fit well with the double exponential function as the pulse interval changes (Figure 8d). Figure 8e–g show the PSC of the device under 450/808/980 nm, with significant differences in photocurrent indicating wavelength selectivity of the device.

On this basis, Ci et al. [35] optimized the device structure and successfully designed and prepared an all-vdW two-terminal ferroelectric p-n heterojunction (α-In_2_Se_3_/SnSe) optoelectronic neuro-device. Figure 8h shows the effect of ferroelectric polarizations on the band-bending amplitude of heterojunctions. When the direction of the built-in electric field (E_bi_) generated by polarization is anti-parallel to the E_bi_ direction of the heterojunction, the band bending amplitude is small and the photoelectric response is weak. On the contrary, when the polarization of α-In_2_Se_3_ is reversed, the band bending is significant, effectively enhancing the separation of photogenerated carriers and thus improving the photoelectric response. Therefore, based on the ferroelectric effect of α-In_2_Se_3_, the reconfigurable photoelectric response of the device can be achieved by pre-applying gate voltage pulses, as shown in Figure 8i. In addition, through synergistic modulation of the E_bi_ from ferroelectric polarization reconstruction and photo-induced polarization switching, the device achieved remarkable performance, including an ultrahigh PPF index of 457% (Figure 8j) and STP-LTP transition (Figure 8k). These exceptional metrics highlight the integration and functional advantages of this two-terminal architecture, offering novel insights for constructing high-precision artificial vision systems.

**Figure 9:**
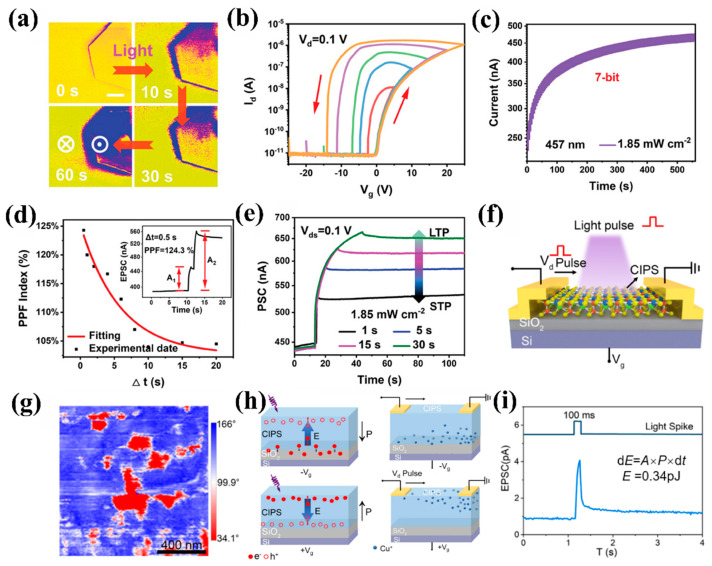
(**a**) PFM test results of the variation of ferroelectric domains with increasing illumination time. (**b**) Transfer characteristics under different gate voltage scanning ranges. (**c**) Multilevel memory characteristics under periodic light pulses. (**d**) PPF index as a function of optical pulse interval time ∆t. (**e**) STP-LTP transition of the devices [98]. (**f**) Schematic of the CIPS-based photoelectric synaptic device. (**g**) Vertical PFM phase mapping of CIPS. (**h**) Schematic representation of photogenerated carrier separation and Cu^+^ ion migration under gate voltage application. (**i**) Energy consumption of devices under a single light pulse. Reprinted with permission from [96]. Copyright 2024 American Chemical Society.

As a widely studied ferroelectric semiconductor, CIPS has been employed by Wang et al. [98] in an all-2D vdW heterostructure (SnS_2_/h-BN/CIPS)-based ferroelectric phototransistor (Fe-FET). As shown in Figure 9a, the polarization switching of CIPS can be cooperatively controlled by light stimulation (457 nm, 5 mW/cm^2^). Benefiting from the unique 2D structure and exceptional optoelectronic response characteristics of CIPS, as illustrated in Figure 9b,c, the device exhibits outstanding performance, such as an ultrahigh current on/off ratio (>10^5^) and 128 distinguishable multilevel conductance states (7-bit storage precision). These characteristics are directly attributed to the ferroelectric domain controllability of CIPS and charge coupling in the CIPS/SnS_2_ interface. The device successfully simulated biological synaptic plasticity (such as PPF, STP, and LTP) and retinal light adaptation behavior through regulation of the polarization of CIPS (Figure 9d,e).

Shang et al. [96] reported a three-terminal optoelectronic synaptic transistor device based on single-layer ferroelectric semiconductor material CIPS (Figure 9f), which achieved synaptic plasticity simulation under optical/electrical dual-mode stimulation through gate-voltage-controlled ferroelectric polarization switching. The regulating effect of gate voltage on ferroelectric domain flipping and carrier separation was confirmed through PFM (Figure 9g). The schematic diagram of photogenerated carrier migration and Cu^+^ ions migration under different gate voltages in CIPS is shown in Figure 9h. The device achieved biological synaptic behaviors under appropriate read voltages, such as PPF, STP, and LTP. By utilizing the ferroelectric ion coupling properties of CIPS, the device demonstrated significantly enhanced synaptic performance through gate-induced in-plane Cu^+^ ions migration and photogenerated carrier separation. Figure 9i presents the energy consumption results for this device, with energy consumption as low as 0.34 pJ per event. These research results demonstrate the enormous potential of ferroelectric CIPS in neural morphology applications, providing new ideas for the application of 2D van der Waals ferroelectric materials in neural morphology calculations.

### 3.3. Polymer Ferroelectric Materials

Polymer ferroelectric materials demonstrate unique advantageous characteristics in the field of neuromorphic devices. From the perspective of electrical performance, this type of material has excellent insulation properties (resistivity usually reaches 10^15^–10^17^ Ω·cm), enabling ultralow energy consumption down to the sub-picojoule (usually ranges from 0.1 to 1 pJ) level during synaptic weight updates via gate-field-controlled polarization switching [99,100,101]. This outstanding energy efficiency originates from their extremely low leakage current density (<10^−9^ A/cm^2^). From the perspective of mechanical properties, ferroelectric polymers represented by PVDF-based polymers possess remarkable mechanical flexibility (elastic modulus ~2–4 GPa, elongation at break > 50%), maintaining stable ferroelectric performance even under repeated bending (radius of curvature < 1 mm) and complex deformations [101,102,103]. This unique flexibility makes it an ideal choice for developing wearable neuromorphic devices that can be seamlessly integrated into flexible electronic systems or biological interface devices.

**Figure 10:**
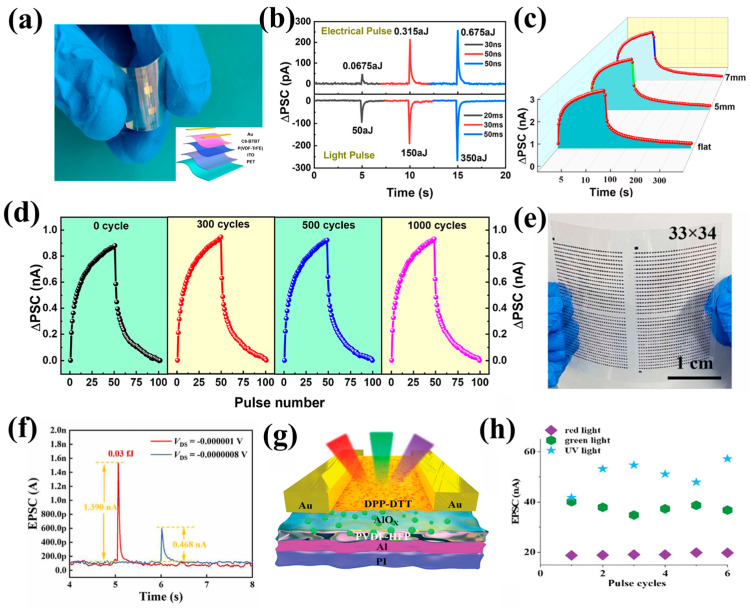
(**a**) Photograph of the flexible optoelectronic device. The inset is the schematic diagram of the device. (**b**) The power consumption of the device under modulation of electrical pulses (top image) and optical pulses (bottom image). Optoelectronic characteristics of devices under different (**c**) bending radii and (**d**) bending periods. Reprinted with permission from [40]. Copyright 2022 American Chemical Society. (**e**) Physical image of large-area (33 × 34) flexible carbon nanotube synaptic photogating transistor array prepared by gravure printing. (**f**) Ultra-low power consumption of the device under a single pulse [36]. (**g**) Structure of flexible synaptic transistor devices. (**h**) EPSC under different wavelength light cycling pulses [104].

Li et al. [40] developed a wearable synaptic transistor based on polymer ferroelectric material P(VDF-TrFE), as shown in Figure 10a. By precisely controlling the rapid switching of β-phase ferroelectric domains in P(VDF-TrFE) with a response time of 30 ns, the device demonstrates ultra-low power consumption characteristics under optical/electronic dual-mode regulation (Figure 10b). Optical pulses consume merely 50 aJ per event (at 360 nm wavelength), while electrical pulses achieve an unprecedented 0.0675 aJ per event (at 10 V/30 ns), which is at least one order of magnitude lower than traditional polymer synaptic devices. In addition, the device maintains exceptional stability under mechanical bending conditions, as illustrated in Figure 10c,d. By controlling the remnant polarization modulation of PVDF-TrFE, the device achieved non-volatile storage of synaptic weights and successfully simulated advanced synaptic behaviors such as associative learning.

As shown in Figure 10e, Wang et al. [36] successfully fabricated a large-area 33 × 34 flexible carbon nanotube synaptic transistor array using roll-to-roll gravure printing technology, employing a composite dielectric layer composed of photosensitive rhodamine 6G (Rh6G) blended with poly(vinylidene fluoride-co-hexafluoropropylene) (PVDF-HFP). The built-in electric field caused by the ferroelectric polarization of PVDF-HFP significantly enhances the separation efficiency of photogenerated carriers, enabling the device to achieve ultralow power consumption (0.03 fJ per pulse event) at low operating voltages (−1.5 V~0.5 V), as shown in Figure 10f. In addition, the device successfully simulated complex biological synaptic functions such as EPSC, PPF, and dynamic learning and forgetting, and it generated high-quality virtual images (7680 × 4096 pixels) through generative adversarial networks (GAN). The PVDF-HFP not only provides a basis for tunable synaptic weight but also enables the transition between LTP/STP through reversible polarization switching, highlighting the exceptional process compatibility and functional scalability of PVDF-HFP in flexible electronics applications.

Wang et al. [104] developed a flexible optoelectronic synaptic transistor utilizing PVDF-HFP with a high HFP content (25 mol%) as the dielectric layer, as shown in Figure 10g. The device leverages the unique ferroelectric polarization and ion migration properties of PVDF-HFP to achieve precise modulation of excitatory/inhibitory postsynaptic currents while exhibiting exceptional mechanical flexibility (with a bending radius as small as 2.5 mm) and ultra-low power consumption (1.2 × 10^−11^ J). Furthermore, the device is capable of multi-wavelength response (365 nm, 525 nm, and 625 nm) by incorporating AlO_x_ nanoparticles into the DPP-DTT matrix to significantly enhance exciton separation efficiency, as depicted in Figure 10h. The above series of works demonstrates the tremendous potential of PVDF-based ferroelectric materials in flexible, multifunctional integrated neuromorphic systems.

## 4. Applications of Ferroelectric-Based Optoelectronic Synaptic Devices

Ferroelectric-based optoelectronic synaptic devices emulate biological synaptic functions at the hardware level, enabling parallel processing, efficient learning, and recognition of massive unstructured data a [26,105,106]. Beyond replicating fundamental synaptic behaviors, these devices have found extensive applications in image recognition and processing, dynamic perception, biological behavior simulation, and optical logic operations [107,108]. This section focuses on exploring the application scenarios of ferroelectric-based optoelectronic synapses in neuromorphic systems.

### 4.1. Image Recognition and Processing

**Figure 11:**
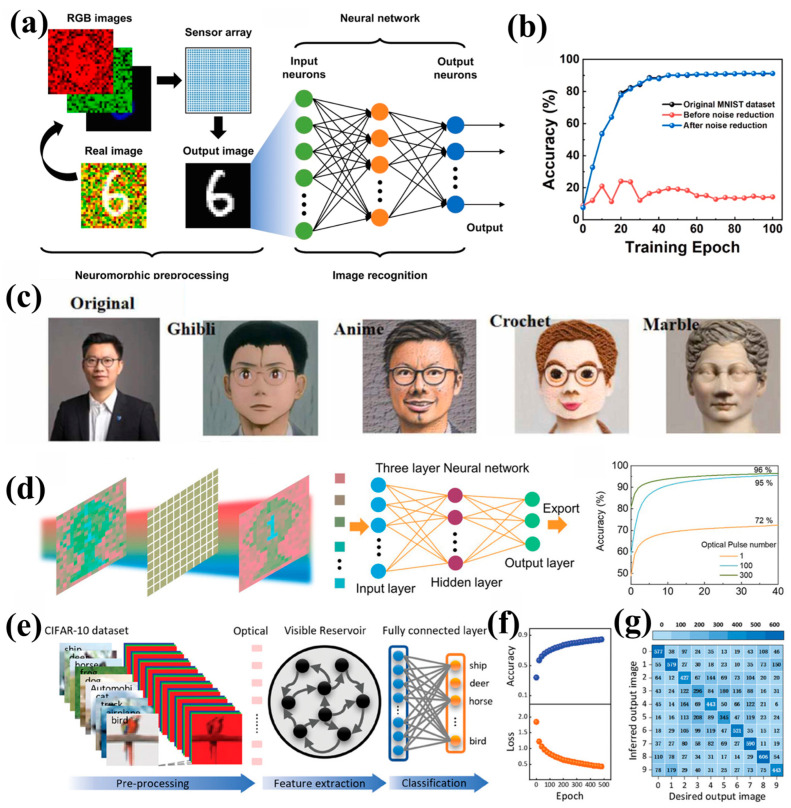
(**a**) The preprocessing of the image noise reduction utilizing the sensor array. (**b**) Comparison of recognition accuracy between images processed differently and the original image [37]. (**c**) Deep integration of virtual reality for generating different styles (Ghibli, anime, crochet, and marble) of characters [36]. (**d**) Image recognition scheme using ANN and comparison of image recognition rates based on different numbers of light pulses [110]. (**e**) Schematic diagram of optical Reservoir Neural Network for Color Image Recognition. (**f**) Classification accuracy and loss rate as a function of training epochs. (**g**) Comparison between experimental classification results and expected output results of the system based on optical hetero-synapses [62].

Image recognition and processing represent core application directions of optoelectronic synapse devices. The functions of optoelectronic synapse devices have gradually expanded from basic digital recognition to multispectral image processing in complex scenes [109,110,111,112]. For example, Du et al. used a neural morphology preprocessing process based on the MoS_2_/BaTiO_3_ synapse sensor array to reduce redundant data, enabling the recognition rate on the MNIST handwritten digit set to significantly increase from 15% to 91% (Figure 11b) [37]. The precise recognition of complex color images with added red and green (R&G) Gaussian noise signals can also be achieved, as shown in Figure 11a, highlighting the advantage of ferroelectric materials in handling redundant data. In addition, a flexible synaptic array based on PVDF-HFP/carbon nanotubes has achieved high-quality virtual image generation of 7680 × 4096 pixels by embedding the LTP/LTD curve of the device into a generative adversarial network (GAN) [36]. The GAN embedding the synaptic plasticity of the transistor during the iterative weight updating process has the advantages of developing the virtual reality and contributes to the integration of the metaverse and neuromorphic computing. Figure 11c illustrates the different styles of characters generated by deep integration of virtual reality. The realism of the generated images reaches a level indistinguishable to the human eye from real images.

In the field of image processing, Li et al. [113] constructed a 3 × 3 pixel array platform based on α-In_2_Se_3_ ferroelectric semiconductor, which achieved a wide spectral response from visible light to short-wave infrared through wavelength-dependent resistance modulation. Combined with the dynamic switching between volatile/non-volatile modes of the device under gate voltage regulation, this work provides a hardware foundation for multispectral image preprocessing. The ReS_2_/P(VDF-TrFE) device array achieves wavelength-selective target extraction in noisy environments through ferroelectric modulation and photoconductive effects, improving recognition accuracy from 72% to 96%, as shown in Figure 11d [110]. In addition, the device successfully simulated the color resolution mechanism of the human retina. Under alternating pulsed stimulation with blue (450 nm) and green (532 nm) light, the device enables differential responses to distinct optical wavelengths via reversible ferroelectric polarization switching, thereby separating target wavelength information from mixed optical signals.

Furthermore, the full vdW heterojunction synapse (WSe_2_/CIPS) responds to visible spectra within a single pixel without the need for a filter through a dual functional layer design, as shown in Figure 11e [62]. By combining the polarization memory effect of CIPS (with a remnant polarization of 6 μC/cm^2^) and the photogenerated charge trapping of WSe_2_, the device achieved an accuracy of 84.9% in color image classification on the CIFAR-10 dataset (Figure 11f,g), while the power consumption of the optical communication system was only 0.4 nW. This enables in-situ hierarchical processing of RGB primary colors, thereby accomplishing edge enhancement and feature extraction for complex images.

### 4.2. Dynamic Perception

**Figure 12:**
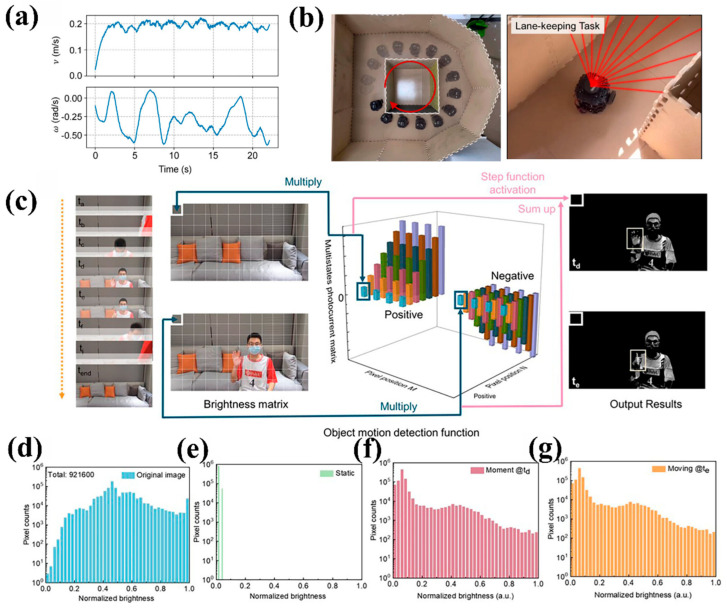
(**a**) The changes calculated in linear and angular velocity vary when the car bypasses a given map. (**b**) Actual diagram of the lane-keeping task. Reprinted with permission from [114]. Copyright 2024 American Chemical Society. (**c**) Object motion detection operation based on the reconfigurable neuromorphic vision sensor. (**d**) Brightness distribution in the original image. After motion detection, the output brightness distribution of (**e**) static image, (**f**) dynamic image at t_d_, and (**g**) dynamic image at t_e_. Reprinted with permission from [28]. Copyright 2024 American Chemical Society.

Dynamic perception is crucial in intelligent perception systems, and innovative designs based on ferroelectric optoelectronic synapse devices provide the possibility for achieving efficient dynamic target capture. Zhou et al. [114] designed a transistor synaptic device based on a 2D ferroelectric semiconductor α-In_2_Se_3_, which achieved perception-and-computing-in-memory (PCIM) function through ferroelectric-optoelectronic coupling characteristics, and applied it to reservoir computing (RC) systems. In the lane-keeping task of autonomous driving, the system obtains real-time obstacle distance information through lidar, directly converts optical signals into synaptic conductivity changes (Figure 12a), and processes dynamic temporal signals using the nonlinear dynamics and short-term memory characteristics of RC architecture. As shown in Figure 12b, the steering commands output by the system enabled smooth obstacle avoidance in the vehicle trajectory, with energy consumption approximately 10^4^ times lower than traditional GPUs and significantly improved data throughput. This achievement highlights the high efficiency and low-power advantages of α-In_2_Se_3_ synaptic devices in dynamic target recognition.

As shown in Figure 12c, Dang et al. [28] simulated the selective response of retinal ganglion cells to motion information using a WSe_2_/P(VDF-TrFE) device, achieving bio-inspired dynamic target detection through ferroelectric modulation. The programmable polarization states of the ferroelectric copolymer enabled the device to exhibit multilevel, uniform bidirectional photocurrent (positive/negative weight) step changes (6 pA). Leveraging this property, the device performed differential calculations on brightness pixels across consecutive frames. Figure 12d depicts the brightness distribution in the original image. Static backgrounds were canceled out due to unchanged luminance (output near zero), while dynamic targets were highlighted due to brightness variations (Figure 12e–g). The nonvolatile multistate storage under ferroelectric modulation (retention time > 10^4^ s) allowed the device to distinguish self-motion (e.g., camera shake) from genuine target motion, retaining only dynamic information for subsequent processing. These works collectively demonstrate that the polarization flip and polymorphic storage characteristics of ferroelectric materials provide a hardware foundation for dynamic visual processing.

### 4.3. Biological Behavior Simulation

**Figure 13:**
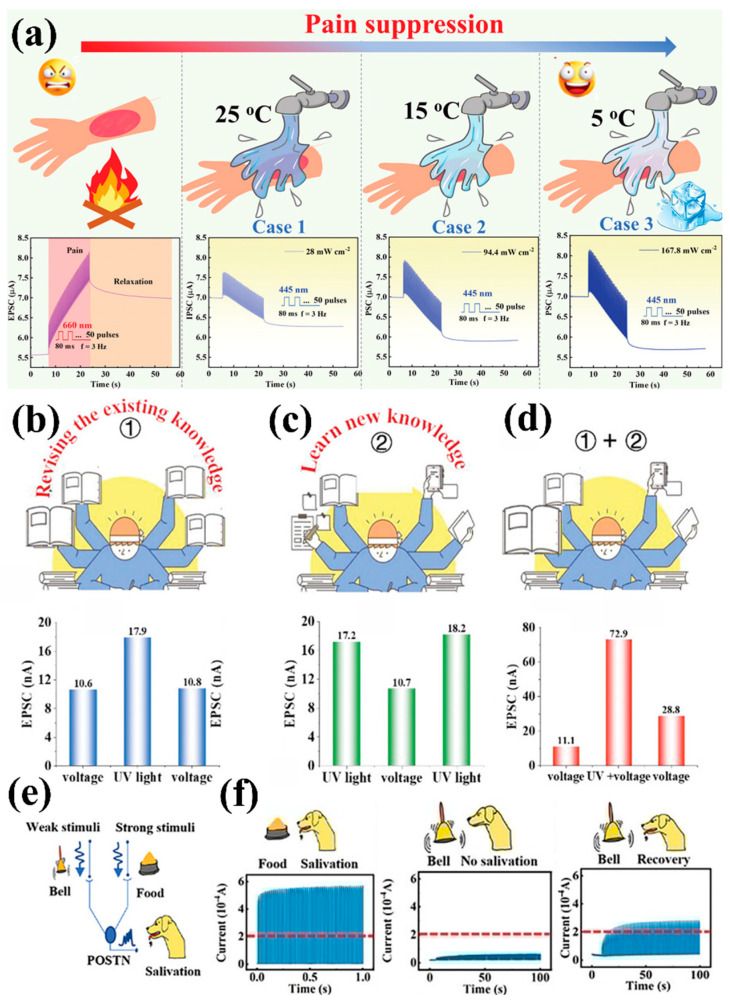
(**a**) Experimental emulation for pain formation and suppression processes of nociceptors [117]. (**b**) Voltage-light-voltage excitation simulation only reviewing old knowledge. (**c**) Light-voltage-light excitation simulation only learns new knowledge. (**d**) Voltage-voltage/light (co-stimulation)-voltage review old knowledge and learn new knowledge at the same time [104]. (**e**) Pavlov’s dog associative learning. (**f**) Simulation of device behavior under “Food” stimuli (electrical pulses) and “Bell” stimuli (optical pulses) [38].

Behavioral simulation (including learning behavior simulation, pain perception, etc.) refers to the reproduction of complex biological neural functions through biomimetic mechanisms, which holds significant importance in artificial intelligence systems [115,116]. For instance, when the human body receives dangerous stimuli, sensory neurons trigger neural alarm signals through cascading action potentials. Therefore, it is crucial to simulate the human capacity for external signal perception using synaptic devices. Ji et al. [117] used CuPc/P(VDF-TrFE) heterojunction devices to simulate action relaxation behavior under nociceptive stimuli. When exposed to external high-temperature stimuli (such as fire), the human body will feel pain. This type of pain usually gradually subsides over time, and rinsing with cold water can accelerate the relief of pain. As shown in Figure 13a, light pulses of 660 nm and 445 nm wavelengths were employed to simulate the enhancing thermal signal and inhibitory cold signal, respectively. When 50 consecutive 660 nm light pulses were applied, the EPSC significantly increased and slowly decayed after removing the light stimulation, effectively simulating the pain behavior of the human body after external stimulation. Subsequently, 50 consecutive 445 nm light pulses of varying power density were applied to simulate the pain relief process using cold water at different temperatures. Higher power density of the light corresponded to lower-temperature cold water stimulation, which reduced the EPSC more rapidly and achieved more pronounced relief effects.

In addition to single light stimulation, the synergistic combination of optical and electrical stimulation can also achieve biomimetic behavior simulation. For example, Wang et al. [104] simulated an efficient learning efficiency model by designing flexible synaptic transistors. The results indicate that the EPSC changes of the device can be almost ignored by alternating voltage-light-voltage stimulation (from 10.6 nA to 10.8 nA), and this process is defined as reviewing old knowledge (Figure 13b). When stimulated alternately by light-voltage-light, the EPSC showed only a slight increase (from 17.2 nA to 18.2 nA, respectively), which can represent the process of only learning new knowledge (Figure 13c). In contrast, alternating voltage-voltage/light (co-stimulation)-voltage significantly enhanced synaptic responses (EPSC increased from 11.1 nA to 28.8 nA), as shown in Figure 13d. This phenomenon can be analogized to the human learning mode of improving learning efficiency by reviewing previous knowledge (electrical stimulation) while acquiring new knowledge (light stimulation).

Furthermore, synaptic devices can simulate classical conditioning behavior, where actions can be modified or learned based on stimuli and responses. A well-known example of such behavior simulation is Pavlov’s dog experiment [118,119]. For instance, Han’s team achieved EPSC enhancement under optical stimulation alone by alternately training the device with light and electrical pulses, mimicking the associative learning of “bell sound induced salivation” [38]. As shown in Figure 13e, electric pulses (1 V, 0.01 s) can independently trigger high currents (>0.2 mA, threshold) for simulating food stimuli; the light pulse (405 nm) initially produces only a weak current (<0.2 mA) for simulating ringtones. By alternately applying electrical and optical pulses for joint training, the polarization characteristics of the photoferroelectric material are utilized to enhance the light response. As the training time increases (400–600 s), the ferroelectric domains gradually align in a preferred orientation, improving photogenerated carrier separation efficiency. This led to a progressive increase in current under optical stimulation alone. After 600 s of training, the optically induced current exceeded the threshold. As shown in Figure 13f, the device successfully associative learning between light and electrical stimuli and effectively replicates a dog’s conditioned reflex to the bell.

In summary, behavioral simulation can effectively reproduce biological behaviors such as pain perception, learning, and association through precise design of optical/electrical signals (such as wavelength, frequency, and polarization) and synergistic optimization of material properties (such as ferroelectricity and carrier separation efficiency).

### 4.4. Optical Logic Operations

**Figure 14:**
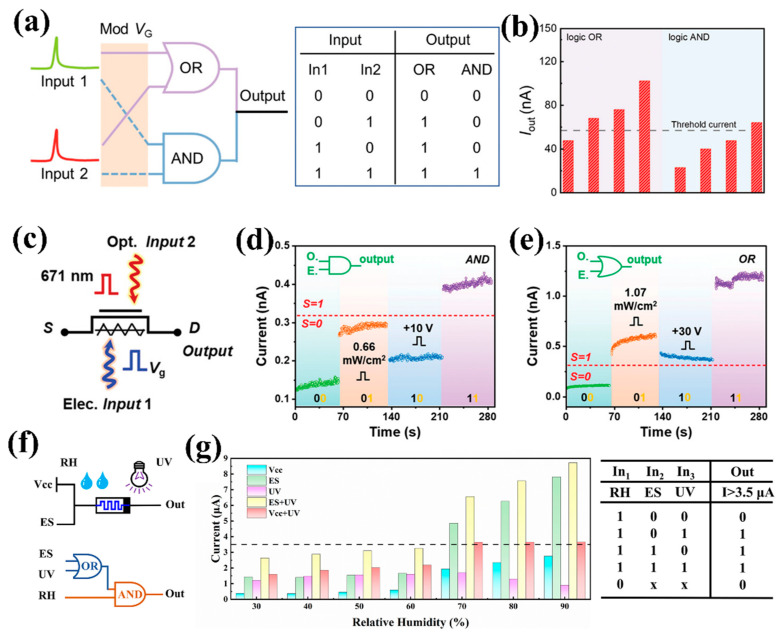
(**a**) Schematic operation diagram of adjustable logic of “AND” and “OR” by using 532 and 808 nm signals as inputs 1 and 2, and using gate voltage as modulatory input. (**b**) Realization logic operations from “OR” to “AND” in output current [22]. (**c**) Schematic operation diagram for the logic functions with electrical and optical inputs in the SnS_2_/In_2_Se_3_ synaptic transistor. (**d**) “AND” and (**e**) “OR” logic operations of the device [39]. (**f**) Logic gate circuit diagram for the three-person voting device. (**g**) Output result of MXene/Y:HfO_2_ memristor running a three-person voter logic function. Reprinted with permission from [123]. Copyright 2024 American Chemical Society.

Optical logic operations enable efficient and low-power information processing while providing the possibility for multimodal signal integration in complex environments [120]. At present, various logic behaviors have been simulated using ferroelectric-based optoelectronic synapse devices [121,122]. Wang et al. [22] utilized the switchable ferroelectric polarization of LiNbO_3_ and collaboratively modulated the carrier distribution in the MoS_2_ channel using dual-wavelength optical pulses, achieving dynamic switching of logic behaviors in a single device. As shown in Figure 14a, 532 nm and 808 nm light pulses were respectively defined as In1 and In2, with the digital signals “0” and “1” representing the “off” and “on” states of the light. For the output signal, a current exceeding the threshold (57 nA) was defined as “1”, otherwise as “0”. Figure 14b describes the output current of logic operations from “OR” to “AND”. When a negative gate voltage was applied, the output reached “1” only when both input signals were triggered simultaneously, realizing an “AND” logic function. Without applying a negative gate voltage, either In1 or In2 could trigger an output current surpassing the threshold current, demonstrating an “OR” logic function.

Optical/electric co-stimulation can also be used as input signals to implement the logical behavior of devices. Ci et al. [39] achieved reconfigurable logic gates in the α-In_2_Se_3_/SnS_2_ heterojunction by introducing optical/electric cooperative control. As illustrated in Figure 14c, Vg pulses of different amplitude voltages and light pulses of varying intensities were used as electric (Input 1) and optical (Input 2) input signals, respectively. The source-drain current served as the output, and a threshold current (0.32 nA) is set to distinguish between logic “1” (above threshold) and “0” (below threshold). When using a 10 V electric pulse and a 0.66 mW/cm^2^ light pulse as input signals, the device functioned as an “AND” logic gate (Figure 14d). When using a 30 V electric pulse and a 1.07 mW/cm^2^ light pulse as input signals, the device operated as an “OR” logic gate, as shown in Figure 14e. The underlying mechanism lies in polarization-induced charge modulation of the interfacial barrier height, combined with the transient modulation of the interface potential barrier by photogenerated carriers, enabling dynamic switching of logic functions within a single device.

In addition to electrical and optical stimulation, new logic devices can also exhibit different logic functions based on changes in temperature or humidity. As shown in Figure 14f, Fang et al. [123] introduced humidity, electrical, and optical as variables to innovatively simulate a “three-person voting” logic system based on MXene/Y:HfO_2_ memristor. The device reading voltage is set to 0.1 V, and the relative humidity, electrical stimulation, and light stimulation abbreviated as RH, ES, and UV, respectively. When two light inputs (representing “approval”) coexisted with high humidity (>60% RH, simulating “veto power”), humidity-induced interface ion migration will destroy the conductive filament and force a low-level output (Figure 14g). This multiphysics field regulation is achieved through the humidity sensitivity of oxygen vacancies in Y:HfO_2_ and the hydrophilicity of MXene, expanding the dimensions of logical behavior. In summary, these works have gradually expanded the logical functions of ferroelectric-based optoelectronic synapse devices from dual light control and optoelectronic collaboration to multimodal integration, providing efficient and flexible hardware solutions for biomimetic vision systems and neural morphology computing.

## 5. Summary and Outlook

The non-volatile polarization strength of ferroelectric materials can be effectively controlled by external fields (such as light, electric fields, humidity, etc.), providing a foundation for the construction of programmable optoelectronic synapse devices and demonstrating great potential in the field of integrated sensing-storage-computing systems. In this review, we introduced the basic functions of synapses and the validation methods in artificial synaptic devices, firstly. From the structural perspective, the advantages and disadvantages of the three-terminal and two-terminal device architectures were summarized separately. From the perspective of materials, this review provides a detailed introduction to the effects of different ferroelectric materials on the characteristics of optoelectronic synapses, including oxide ferroelectrics, two-dimensional ferroelectrics, and polymer ferroelectrics. Finally, the specific applications of optoelectronic synapse devices in image recognition and processing, dynamic perception, biological behavior simulation, and optical logic operations were introduced. While significant progress has been made in ferroelectric-based optoelectronic synapses with promising application prospects, the current research is still in the process of development and facing multiple challenges in the future development process.

At the material level, by constructing ferroelectric materials with nanodomain or topological domain structures and optimizing domain size and dynamic properties, synapses can be endowed with the ability to regulate behavior at multiple time scales. This can not only simulate the dynamic information processing mechanism of biological synapses more accurately but also largely overcome the size scaling effect of devices. Meanwhile, for ferroelectric/semiconductor heterojunctions, introducing suitable inert buffer layers can enhance the interface stability of heterojunctions. For 2D ferroelectrics, excellent interfacial contact can be achieved through van der Waals bonding. The preparation of large-area uniformity of 2D ferroelectrics is also one of the challenges faced in this field. Furthermore, the exploration of ferroelectric materials sensitive to multi-physical fields such as temperature, humidity, and magnetic fields is expected to overcome the limitations of traditional optical/electrical polarization regulation, enabling multi-modal coupled regulation control of devices.

At the device level, the combination of flexible architecture and low-power (nW level) technology will enhance the compatibility between devices and biological systems. In addition, most of the reported optoelectronic synapses can only operate within a relatively narrow spectral range, which inevitably limits their application in the neuromorphic visual system over a wide spectral range. At present, most research achieves wideband response of devices by modulating the bandgap of semiconductor materials. In fact, ferroelectric materials can also achieve sensing of light signals in specific wavelengths, significantly broadening the response wavelength of the device. For instance, employing pyroelectric ferroelectric materials as gate dielectrics could enable effective infrared-band regulation of semiconductor channels, thereby dramatically expanding the operational spectrum to meet complex environmental demands.

At the application level, it is necessary to shift from studying the characteristics of single synapses to integrating multiple neural networks. By simulating the human brain’s information transfer mechanisms, this approach can enable advanced cognitive functions (such as temporal prediction). While driving the development of bio-inspired vision algorithms and chip systems to achieve practical applications in autonomous vehicles and robotics. In addition, the integration of flexible optoelectronic synapse devices with wearable systems will provide innovative solutions for applications such as real-time health monitoring.

It can be confidently anticipated that continued innovations across materials, devices, and algorithms will propel optoelectronic synapses from laboratory research to diverse application scenarios such as multimodal sensing networks and intelligent Internet of Things. These advancements will serve as the core driving force behind performance breakthroughs in neuromorphic computing and artificial intelligence, ultimately ushering in a new era of brain-inspired intelligent technologies.

## Data Availability

No new data were created or analyzed in this study.
